# Conium

**Published:** 1897-01

**Authors:** 


### HOMŒOPATHIC PHYSICIAN,

A MONTHLY JOURNAL OF

HOMŒOPATHIC MATERIA MEDICA AND CLINICAL MEDICINE.

"If our school ever gives up the strict inductive method of Hahnemann, we are lost, and deserve only to be mentioned as a caricature in the history of medicine."—CONSTANTINE HERING.

Vol. XVII. JANUARY, 1897. No. 1.

### EDITORIAL.

CONIUM.—Comparatively few notes from Dr. Lippe's lectures upon Conium have been obtained by the editor, and so he is unable to give any extended comparisons unless he were to copy extensively from his own note-book. But that is not the purpose of these editorials. As frequently stated, the purpose has been to reproduce as much as possible from Dr. Lippe's lectures the comments made by him, and which the editor was fortunate enough to record.

One of the most remarkable of the symptoms of Conium is the vertigo. The patient is attacked with vertigo when lying in bed. If he turn to one side or the other he has vertigo as if the bed were floating. This is, indeed, the keynote of Conium. It is an old and reliable indication for Conium, and must be well known to most of our readers. It resembles Silicea, which has vertigo when turning the head *to the left* while lying down. A distinguished lady, who had done much to advance the cause of Homœopathy, sent for the writer to treat her for a vertigo of this character. She had to lie very still on a couch with the head turned somewhat to the right. Any attempt to turn it to the left would cause a violent vertigo and the impression that the walls and ceiling were falling in upon her. Though perfectly well aware that it was a delusion, she could not help uttering a little scream. The editor gave her

Silicea$^{200}$, and brought about prompt relief. In speaking of it afterward she said: "While I have always understood the great possibilities of Homœopathy, I certainly was much astonished at the quickness and permanence of the relief I experienced in this case."

The Conium vertigo is a sense of floating of the bed when turning the head to either side. A distinguishing characteristic of Conium is the intoxication from small quantities of wine. In commenting upon this symptom, Dr. Lippe said that the law of the similars ought to aid us in selecting the proper wine for certain patients to whom it was a benefit. In the Rhine countries we are impressed with the large number of cases of stone in the bladder. There appear to be more of these cases in that region than anywhere else in the world. In these districts hock wine is produced, and so we find hock wine a good remedy in certain cases of gout. Hungarian wines contain small portions of Phosphorus. Hungarian wines are useful for people who are exhausted by severe brain work. Burgundy also is a good wine for people exhausted by too much brain work.

Whisky will cause acidity of the stomach. Whisky drinkers sometimes die of cancer of the pyloric end of the stomach. Dr. Lippe remembered a case of cancer of the stomach greatly relieved by a teaspoonful of whisky at dinner. People who are troubled with acidity of the stomach, and who have not received benefit from medicine, have been cured by a tablespoonful of whisky at dinner.

Madeira wine will cause palpitation of the heart. For those who suffer from heart affections Madeira is the proper wine to drink. Madeira has a wonderful effect in such cases. Madeira is also a good wine to be taken by gouty people. To prescribe a wine properly we ought to learn its sick-making power.

Some persons have a longing for champagne. In such cases the desire should be gratified. A small quantity will sometimes enable a patient to turn a corner, so to speak, toward recovery.

Wines must, of course, be prescribed in very small quantities to do good to those for whom they are indicated.

Conium has intoxication from small quantities of wine, and Cactus-grandifloris and Zinc have headache from small quantities of wine.

The following comparisons are added by the editor, some obtained from Dr. Lippe and some from other sources:

Rhododendron has pain in forehead and temples when lying in bed, made worse by drinking wine.

Tabaccum has sensitiveness to the smell of wine. The fumes of it almost intoxicate.

Cactus-grand. and Zinc have headache from wine. Zinc has headache from even the smallest quantities of wine.

Bovista, Conium, and Rhododendron have intoxication from small quantities of wine.

Belladonna increase of dyspnœa from wine.

Gelsemium, eye-symptoms made worse from wine.

Silicea, wine in small quantities causes ebullitions and thirst.

Bryonia, heart-burn from a glass of wine in the evening.

Kali-muriaticum, wine and beer intoxicate easily.

